# Gestational Hormone Concentrations Are Associated With Timing of Delivery in a Fetal Sex-Dependent Manner

**DOI:** 10.3389/fendo.2021.742145

**Published:** 2021-09-15

**Authors:** Amber L. Cathey, Deborah J. Watkins, Zaira Y. Rosario, Carmen M. Vélez Vega, Bhramar Mukherjee, Marie S. O’Neill, Rita Loch-Caruso, Akram N. Alshawabkeh, José F. Cordero, John D. Meeker

**Affiliations:** ^1^Department of Environmental Health Sciences, University of Michigan School of Public Health, Ann Arbor, MI, United States; ^2^Graduate School of Public Health, University of Puerto Rico, San Juan, PR, United States; ^3^Department of Biostatistics, University of Michigan School of Public Health, Ann Arbor, MI, United States; ^4^Department of Epidemiology, University of Michigan School of Public Health, Ann Arbor, MI, United States; ^5^College of Engineering, Northeastern University, Boston, MA, United States; ^6^College of Public Health, University of Georgia, Athens, GA, United States

**Keywords:** birth cohort, corticotropin releasing hormone, gestational age, pregnancy, preterm birth, progesterone

## Abstract

**Background:**

Early delivery remains a significant public health problem that has long-lasting impacts on mother and child. Understanding biological mechanisms underlying timing of labor, including endocrine disruption, can inform prevention efforts.

**Methods:**

Gestational hormones were measured among 976 women in PROTECT, a longitudinal birth cohort in Puerto Rico. We evaluated associations between hormone concentrations at 18 and 26 weeks gestation and gestational age at birth, while assessing effect modification by fetal sex. Exploratory analyses assessed binary outcomes of overall preterm birth (PTB, <37 weeks gestation) and the spontaneous PTB subtype, defined as preterm premature rupture of membranes, spontaneous preterm labor, or both. Multivariable logistic and linear regressions were fit using visit-specific hormone concentrations, and fetal sex-specific effects were estimated using interaction terms. Main outcome models were adjusted for maternal age, education, marital status, alcohol consumption, environmental tobacco smoke exposure, and pre-pregnancy body mass index (BMI). Exploratory models adjusted for maternal age and education.

**Results:**

We observed reduced gestational age at birth with higher circulating CRH (β: -2.73 days, 95% CI: -4.97, -0.42), progesterone (β: -4.90 days, 95% CI: -7.07, -2.73), and fT4 concentrations (β: -2.73 days, 95% CI: -4.76, -0.70) at 18 weeks specifically among male fetuses. Greater odds of overall and spontaneous PTB were observed among males with higher CRH, estriol, progesterone, total triiodothyronine (T3), and free thyroxine (fT4) concentrations. Greater odds of PTB among females was observed with higher testosterone concentrations.

**Conclusions:**

Various associations between hormones and timing of delivery were modified by fetal sex and timing of hormone measurement. Future studies are needed to understand differential mechanisms involved with timing of labor between fetal sexes.

## Introduction

Delivery before 37 weeks of gestation, or preterm birth (PTB), is the leading cause of neonatal mortality worldwide (1). The preterm birth rate in the United States is higher than that of most other high-income nations and increased to 9.85% from 2014 to 2016 (2). This increase was primarily driven by late PTBs, which occur between 34 and 36 weeks gestation, suggesting that development of targeted prediction strategies for detecting pregnancies destined for late preterm delivery could have marked impacts on the public health burden of early delivery. Infants born preterm are at increased risk for adverse health outcomes later in life including reduced renal function (3), neurodevelopmental impairments (4), cerebral palsy (5), and reduced myocardial function (6). Despite being a common public health problem, the causes of early delivery are largely unknown.

Preterm birth can be subcategorized into spontaneous and indicated groups based on obstetric presentation, and some have suggested that assessing these subtypes separately may better inform their etiologies. The spontaneous subtype occurs from spontaneous premature initiation of labor or rupture of membranes, and is characterized by an inflammatory uterine environment which is not present in medically indicated PTBs (7). The physiological changes that occur upstream of clinical detection of PTB may be distinct between subgroups and could help clinicians determine pregnancies that are at high risk for early delivery.

The maternal and fetal endocrine milieus change and interact in unique ways at different points throughout gestation. Numerous hormones play critical roles during pregnancy and so understanding their effects on the uterine environment and fetal growth could shed light on mechanisms involved with early delivery. Hormones of special interest during pregnancy can be broken into two categories: reproductive hormones and thyroid hormones. Reproductive hormones include testosterone, sex hormone binding globulin (SHBG), estriol, and progesterone. Elevated concentrations of testosterone are observed in women with polycystic ovarian syndrome (PCOS), as well as women with recurring miscarriages. Testosterone may reduce levels of endometrial secretory proteins which are positively associated with length of gestation (8), or it may act antagonistically with estrogens (9). Testosterone circulates through the body bound to SHBG, so changing SHBG concentrations could affect the concentration of bioavailable testosterone. The coordination of concentrations of estriol and progesterone are critically important for the timing of labor, as estriol primes the uterus for contractions (10) and progesterone promotes quiescence of the uterus until the time of labor (11, 12). Progesterone keeps the actions of estriol in check through pregnancy, but an untimely shift in dominance from progesterone to estriol could result in early delivery.

Thyroid hormones are critical early in pregnancy for proper brain and skeletal development of the fetus (13). The maternal supply of thyroxine (T4) is particularly important in the first half of pregnancy, before the fetal thyroid gland has matured enough to produce adequate hormones (14). Through gestation, thyroid hormones are important for fetal growth and are correlated with infant weight and length at birth (15). Previous studies have demonstrated associations between clinical hyper- and hypothyroidism and adverse birth outcomes (16–19), but much less is known about subclinical thyroid disruption and gestational length.

Finally, corticotropin releasing hormone (CRH), which is secreted from the hypothalamus and normally involved in stress response, may also be key in understanding the endocrine role in early delivery. CRH concentrations are low in the first half of pregnancy and then begin to exponentially increase around the 20^th^ week of gestation to peak at birth (20). An earlier and more rapid increase of CRH concentrations has been observed in women who experience early delivery (21), suggesting that CRH may be involved in a placental clock and that monitoring CRH concentrations early in pregnancy could provide clues about pregnancies at risk for early delivery.

Few large epidemiological studies exist that assess a wide array of hormone concentrations and timing of delivery. The majority of existing research focuses on one hormone/class of hormones, which makes it challenging to gain a broad understanding of the endocrine pathways implicated in the etiology of early delivery (22–24). Importantly, the spontaneous subtype of PTB has not been well studied. Few previous studies have investigated hormone concentrations at more than one time point during gestation, which is critical because some hormone concentrations change dramatically throughout gestation. Previous work also has not widely assessed the impact of fetal sex on these associations, despite existing literature showing male fetuses conferring greater risk of early delivery, perhaps *via* a greater pro-inflammatory environment (25) or relatively greater weight for gestational age at birth (26). Further, concentrations of hormones such as testosterone, estriol, and progesterone have been shown to be influenced by the sex of the fetus (27–29). Because of these gaps in the literature, the aim of this study was to investigate associations between various hormone concentrations and gestational age at birth, specific to timing of hormone assessment and fetal sex. Additionally, we conducted an exploratory analysis in which binary outcomes of overall PTB and spontaneous PTB were assessed.

## Materials And Methods

### Study Population

Pregnant women were recruited into the PROTECT birth cohort between 2011 and 2018 at 14±2 weeks’ gestation from seven hospitals and prenatal clinics in northern Puerto Rico. Study design and recruitment protocols have been described elsewhere (30). Briefly, women were recruited at 14±2 weeks gestation and were eligible to participate if they were between the ages of 18 and 40 years, participated in their first clinic visit before their 20^th^ week of pregnancy, had not taken oral contraceptives within 3 months of getting pregnant, had not used *in vitro* fertilization to get pregnant, and had no known preexisting medical or obstetric conditions. Demographic and self-reported health information was provided at the first clinic visit. This study was approved by the research and ethics committees of the University of Michigan School of Public Health, University of Puerto Rico, Northeastern University, and participating hospitals and clinics. All methods reported in this study were performed in accordance with relevant guidelines and regulations imposed by those institutions. All study participants provided full informed consent prior to participation.

### Hormone Measurements

All women provided serum samples at their first and third clinic visits, aligning with median 18 (range 16-20) and 26 (range 24-28) weeks gestation which are periods of rapid fetal growth and development. Serum samples were analyzed at the Central Ligand Assay Satellite Services (CLASS) laboratory in the Department of Epidemiology at the University of Michigan School of Public Health. Progesterone, sex hormone-binding globulin (SHBG), testosterone, total triiodothyronine (T3), total thyroxine (T4), free thyroxine (fT4), and thyroid-stimulating hormone (TSH) were measured using a chemiluminescence immunoassay. Estriol and corticotropin releasing hormone (CRH) were measured using an enzyme immunoassay. Some hormone concentrations were not available for all participants due to sample volume limitations. The ratios of progesterone to estriol (Prog/E3) and T3 to T4 (T3/T4) were assessed in addition to measured hormones because of previous research indicating that the ratios may be better indices of gestational age at birth than single hormone measurements (23, 31, 32). All hormone concentrations below the limit of detection (LOD) were replaced by the LOD divided by the square root of two.

### Birth Outcome Assessment

Based on recommendations from the American College of Obstetricians and Gynecologists, self-reported date of the last menstrual period was collected at the first study visit and used in combination with early ultrasound measurements to determine gestational age at birth (33). Briefly, the LMP was used as the gold standard and was compared to ultrasound measurements taken primarily before 14 weeks gestation. Gestational age was changed from the LMP estimate to the ultrasound estimate if the difference between the two methods was greater than a certain number of days, depending on which week the ultrasound was performed. PTB was defined as delivery before 37 weeks gestation. We also assessed spontaneous PTB, defined as PTB presenting with premature rupture of membranes, spontaneous preterm labor, or both (7).

### Statistical Methods

Distributions of demographic, health, and pregnancy characteristics were calculated. Distributions of hormone concentrations were assessed individually at each study visit. Summary measures of gestational hormone concentrations were also assessed for descriptive purposes using arithmetic means of all available concentrations for each study participant, or geometric means for log-normally distributed hormones. Intraclass correlation coefficients (ICCs) were used to assess between- and within-individual variability of hormone concentrations across study visits.

Linear regression was used to model gestational age at birth, and logistic regression was used to model overall and spontaneous PTB. All statistical models utilized visit-specific hormone concentrations as opposed to summary measures. Effect estimates specific to fetal sex and study visit were derived using two interaction terms: one between the exposure variable and an indicator for fetal sex, and one between the exposure variable and an indicator for study visit. Interaction term p-values less than 0.05 were deemed significant. Sandwich estimators were used in these models to correct for biased standard errors due to the non-repeating nature of outcome variables. Possible confounders were first explored by evaluating their associations with exposure and outcome variables. Then, possible confounders were added into statistical models in a forward stepwise manner and retained in the model if the resultant change in the main effect estimate was greater than 10%. Main gestational age at birth models adjusted for categorical forms of maternal age, maternal education, marital status, exposure to environmental tobacco smoke, alcohol consumption, and pre-pregnancy BMI. All models assessing testosterone also included SHBG to adjust for bound testosterone. In exploratory analyses assessing binary outcomes of overall and spontaneous PTB, statistical models were adjusted only for maternal age and maternal education to maximize sample size.

## Results

Demographics of the study population are shown in **Table 1**. The majority of mothers were under the age of 30 (67.1%), had at least some college education (79%), were employed (63%), had an annual household income under $30,000 (63.1%), were married (53.1%), reported never smoking (86%) or being exposed to environmental tobacco smoke (88.7%), did not drink alcohol during pregnancy (93.6%), had given birth to less than 2 previous children (86.9%), and had a pre-pregnancy BMI of less than 25 (56.1%).

**Table 1:** Maternal demographic characteristics of the study population (N=976).

	N (%)
**Maternal Age (years)**	
18-24	354 (36.3%)
25-29	301 (30.8%)
30-34	206 (21.1%)
35-41	115 (11.8%)
**Maternal Education**	
GED or less	203 (21%)
Some College	331 (34.2%)
Bachelors or Higher	433 (44.8%)
**Employment Status**	
No	357 (37%)
Yes	608 (63%)
**Annual Household Income**	
<10k	269 (31.6%)
10k-<30k	268 (31.5%)
30k-<50k	203 (23.8%)
>=50k	112 (13.1%)
**Marital Status**	
Single	197 (20.4%)
Married	521 (53.9%)
Cohabitating	249 (25.7%)
**Smoking Status**	
Never	833 (86%)
Ever	121 (12.5%)
Current	15 (1.55%)
**Daily Environmental Tobacco Smoke Exposure**	
Never	808 (88.7%)
1 Hour or less	40 (4.39%)
>1 Hour	63 (6.92%)
**Alcohol Use**	
Never	504 (52.2%)
Yes, before Pregnancy	400 (41.4%)
Yes, currently	62 (6.42%)
**Number of Previous Children**	
0	355 (42.7%)
1	367 (44.2%)
2 to 5	109 (13.1%)
**Pre-Pregnancy BMI**	
[0,25]	520 (56.1%)
(25, 30]	240 (25.9%)
Above 30	167 (18%)
**Fetal Sex**	
Female	464 (48%)
Male	502 (52%)

Distributions of hormone concentrations are shown in **Supplementary Table S1**. ICCs for most hormones were high (0.65-0.86), indicating only modest variability in hormone measurements within individuals across study visits. Exceptions were estriol (ICC: -0.22, 95% CI: -0.35, -0.11) and progesterone (ICC: 0.07, 95% CI: -0.04, 0.17), both of which displayed significantly higher concentrations at 26 weeks compared to 18 weeks.

Distributions of birth outcomes, stratified by fetal sex, are shown in **Table 2**. The median gestational age at birth was 39.1 weeks for pregnancies carrying either a male or a female fetus. Both overall and spontaneous PTB occurred more frequently among pregnancies with a male fetus (10.7% and 6.6%, respectively) than those with a female fetus (8.9% and 4.9%, respectively).

**Table 2:** Distributions of gestational age at birth, overall preterm birth, and spontaneous preterm birth.

Gestational Age (wks)	Min	10th	25th	50th	75th	90th		Max
Male Fetuses	23.3	36.6	38.3	39.1	40.0	40.7		42.7
Female Fetuses	21.1	37.0	38.1	39.1	40.1	40.7		42.7
	**Male Fetuses**	**Female Fetuses**						
**Preterm Birth**	**N (%)**	**N (%)**						
Yes	53 (10.7%)	41 (8.9%)						
No	444 (89.3%)	419 (91.1%)						
**Spontaneous Preterm Birth**	**N (%)**	**N (%)**						
Yes	32 (6.6%)	22 (4.9%)						
No	451 (93.4%)	428 (95.1%)						

### CRH and Reproductive Hormones

**Figure 1:**
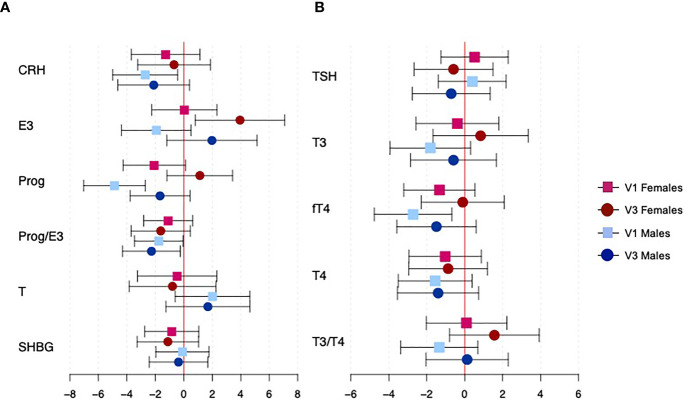
Associations between hormone concentrations and gestational age at delivery, specific to fetal sexes and study visit. Panel **(A)** shows CRH and reproductive hormones, and panel **(B)** shows thyroid hormones. Effect estimates represent the change in gestational age at birth (in days) with an interquartile range increase in hormone concentration. Red and blue estimates correspond to female and male fetuses, respectively. Squares denote effects from hormones at visit 1, while circles denote effects from hormones at visit 3.

**Figure 1A** shows the associations between CRH and reproductive hormone concentrations and gestational age at birth, specific to fetal sex and study visit (effect estimates, confidence intervals, and p-values for fetal sex interaction terms can be found in **Supplementary Table S2**). Fetal sex significantly modified the association between progesterone and gestational age at birth (interaction p=0.015); among male fetuses only, an IQR increase in progesterone at 18 weeks was associated with a decrease in gestational age at delivery of 4.9 days (95% CI: 2.73, 7.07). Other significant findings were observed without significant effect modification by fetal sex. An IQR increase in CRH among male fetuses at 18 weeks was associated with a reduction in gestational age at delivery of 2.73 days (95% CI: 0.42, 4.97). Male fetuses also had a marginal reduction in gestational age at birth with an IQR increase in the ratio of progesterone to estriol at both visits (visit 1 β: -1.75 days, 95% CI: -3.5, -0.07; visit 3 β: -2.24 days, 95% CI: -4.27, -0.28). Finally, female fetuses experienced an increase in gestational age at birth of 3.92 days (95% CI: 0.77, 7.07) with an IQR increase in estriol at 26 weeks.

### Thyroid Hormones

**Figure 1B** shows the associations between thyroid hormone concentrations and gestational age at birth, specific to fetal sexes and study visits (effect estimates, confidence intervals, and p-values for fetal sex interaction terms can be found in **Supplementary Table S2**). We did observe differences in these associations between fetal sexes, though none of them reached statistical significance. Generally, greater reductions in gestational age at birth were observed among male fetuses, but the only finding to reach statistical significance was for fT4 at 18 weeks (β: -2.73 days, 95% CI: -4.76, -0.70).

### Exploratory Analyses of Binary Outcomes

**Figure 2:**
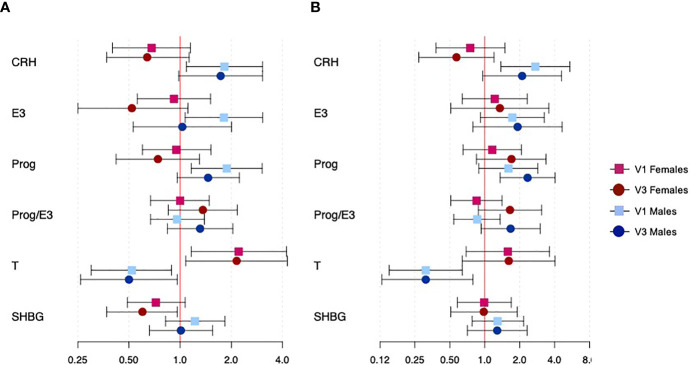
Associations between CRH and reproductive hormone concentrations and binary outcomes of overall and spontaneous PTB, specific to fetal sexes and study visit. Panel **(A)** shows overall PTB and panel **(B)** shows spontaneous PTB. Effect estimates represent the odds of overall or spontaneous PTB with an interquartile range increase in hormone concentration. Red and blue estimates correspond to female and male fetuses, respectively. Squares denote effects from hormones at visit 1, while circles denote effects from hormones at visit 3.

Results for exploratory analyses assessing the binary outcomes of overall and spontaneous PTB and their associations with CRH and reproductive hormones are shown in **Figure 2** (effect estimates, confidence intervals, and p-values for fetal sex interaction terms can be found in **Supplementary Table S2**). Fetal sex effect modification was much more apparent for these outcomes than for continuous gestational age at delivery. The associations between CRH and both overall and spontaneous PTB were significantly modified by fetal sex (interaction p=0.002 and 0.003, respectively); effect estimates were inverse among female fetuses and positive among male fetuses for both outcomes at both study visits, though only effects with hormones at 18 weeks among male fetuses were significant (overall PTB OR: 1.82, 95% CI: 1.09, 3.05; spontaneous PTB OR: 2.73, 95% CI: 1.38, 5.43). Fetal sex also modified the association between estriol and overall PTB (interaction p=0.022); female fetuses saw a marginal reduction in the odds of PTB with an IQR increase in estriol at 26 weeks (OR: 0.52, 95% CI: 0.25, 1.11), while male fetuses saw an increase in odds of PTB with an IQR increase in estriol at 18 weeks (OR: 1.81, 95% CI: 1.07, 3.06). Results for progesterone were similar; fetal sex modified only associations with overall PTB (interaction p=0.011), and significant increased odds of PTB were observed for male fetuses at 18 weeks (OR: 1.88, 95% CI: 1.16, 3.04). Though effect estimates were not significant, study visit was important for determining the associations between the ratio of progesterone to estriol and both overall and spontaneous PTB; effect estimates were positive and larger in magnitude at 26 weeks compared to 18 weeks among both male and female fetuses. Testosterone showed highly significant effect modification by fetal sex for both overall and spontaneous PTB (both interaction p<0.001), with an IQR increase in testosterone conferring lower risk of PTB among male fetuses and higher risk among female fetuses which did not differ between study visits. Female fetuses saw 2.21 times the odds (95% CI: 1.16, 4.23) of overall PTB with an IQR increase in testosterone at 18 weeks, while males saw 0.52 times the odds (95% CI: 0.30, 0.89) at the same study visit. Finally, fetal sex modified the association between SHBG and overall PTB; an IQR increase in SHBG was associated with 0.60 times the odds (95% CI: 0.37, 0.96) of PTB only among female fetuses at 26 weeks.

**Figure 3:**
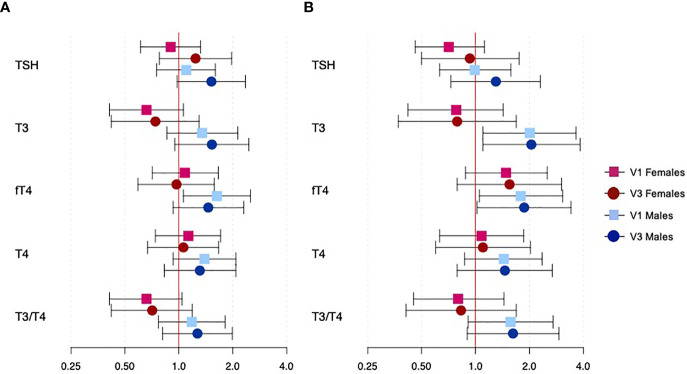
Associations between thyroid hormone concentrations and binary outcomes of overall and spontaneous PTB, specific to fetal sexes and study visit. Panel **(A)** shows overall PTB and panel **(B)** shows spontaneous PTB. Effect estimates represent the odds of overall or spontaneous PTB with an interquartile range increase in hormone concentration. Red and blue estimates correspond to female and male fetuses, respectively. Squares denote effects from hormones at visit 1, while circles denote effects from hormones at visit 3.

Results for exploratory analyses assessing the binary outcomes of overall and spontaneous PTB and their associations with thyroid hormones are shown in **Figure 3** (effect estimates, confidence intervals, and p-values for fetal sex interaction terms can be found in **Supplementary Table S2**). Though most associations were not significant, effect modification by fetal sex was observed for T3 on both overall and spontaneous PTB (both interaction p=0.013), and the ratio of T3 to T4 on overall PTB (interaction p=0.032). An IQR increase in T3 at both study visits resulted in reduced odds of PTB among female fetuses and increased odds of PTB among male fetuses, though only results for spontaneous PTB among male fetuses were significant (visit 1 OR: 2.01, 95% CI: 1.10, 3.65; visit 3 OR: 2.05, 95% CI: 1.10, 3.84). An IQR increase in fT4 at both study visits resulted in marginally increased odds of overall and spontaneous PTB among only male fetuses. Lastly, an IQR increase in the ratio of T3 to T4 resulted in reduced odds of PTB among female fetuses and increased odds of PTB among male fetuses, though none of these associations reached statistical significance.

## Discussion

In the present analysis we explored associations between hormone concentrations measured at two time points during mid-gestation and timing of delivery. Because of previous work indicating dramatic changes in some hormone concentrations through pregnancy, as well as differential susceptibility to adverse pregnancy outcomes based on fetal sex, we determined effect estimates specific to hormones measured at 18 *versus* 26 weeks gestation and male *versus* female fetal sexes. Overall, our results suggest that some hormones, particularly reproductive hormones, have different effects on timing of delivery depending on the sex of the fetus, and most associations show male fetuses being at greater risk for early delivery. We also observed that some associations differed when hormones were assessed at 18 *versus* 26 weeks gestation. An exploratory analysis of the binary outcomes overall and spontaneous PTB revealed similar findings with greater significance. Interestingly, results for the spontaneous subtype of PTB were less affected by study visit than results for overall PTB, supporting separation of these outcomes into more homogenous subgroups.

### CRH and Reproductive Hormones

Reduced gestational age at birth was observed among only male fetuses with increased concentrations of CRH at 18 weeks. While very little epidemiologic work has been done to understand the roles of CRH in adverse birth outcomes, researchers have speculated its influence on labor and delivery for decades. Detection of elevated concentrations of CRH during the second trimester in women who eventually delivered preterm compared to those who delivered after 37 weeks was first described by Campbell et al. in 1987 (34). Since then, CRH has been described as being involved in a “placental clock,” with CRH concentrations as early as 20 weeks possibly setting the stage for future preterm delivery (21). CRH receptors are present in the myometrium (35) and in the fetal zone of the fetal adrenal gland (36), so CRH could exert its effects on labor by interacting with these receptors.

We observed reduced gestational age, and greater odds of overall and spontaneous PTB, with increasing progesterone concentrations when fetal sex was male, but other studies demonstrating similar significant associations are lacking. One study observed progesterone concentrations measured between 28 and 32 weeks gestation to be higher among women who delivered preterm compared to full term (22). We observed higher progesterone concentrations among PTB cases when fetal sex was male, but only around 18 weeks gestation. We also observed higher progesterone concentrations around 26 weeks among women who spontaneously delivered preterm compared to women who carried to term, regardless of fetal sex.

Previous work has shown that a ratio favoring estriol in mid-pregnancy (23) and at delivery (37) is associated with earlier time of labor. Progesterone concentrations rise steadily during pregnancy, contributing to uterine quiescence, downregulation of prostaglandin production, and immune tolerance of the fetus (11, 12). At the onset of human labor, progesterone concentrations do not notably decrease; rather, the body’s response to progesterone is dampened. It is not clear exactly how this occurs, but possibilities include reduction in progesterone receptor expression, changes in receptor isoforms, and local progesterone metabolism (38). As term approaches, the ratio of progesterone to estriol shifts to favor estrogens, with the functional decrease in progesterone driving initiation of labor (39). The new dominance of estrogens promotes an increase in prostaglandin and oxytocin receptors and enzymes responsible for muscle contractions, which work together to help promote labor (10). We observed a positive association between odds of PTB and estriol concentrations when fetal sex was male, but we also unexpectedly observed later gestational age at birth with higher concentrations of estriol at 26 weeks gestation when the fetus was female. In contrast with previous studies, we observed that a higher ratio of progesterone to estriol was associated with reduced gestational age at birth. Interestingly, among women who delivered preterm, a previous study observed a lower ratio of progesterone to estriol among only those without premature rupture of membranes (40), possibly implicating different endocrine pathways in the occurrence of the spontaneous subtype of PTB.

We observed significant effect modification by fetal sex on the associations between testosterone and overall and spontaneous PTB, for which the effect was positive among female fetuses and protective among male fetuses. Previous research on the effects of fetal sex on circulating maternal testosterone has been mixed. Though earlier research suggested that male fetuses conferred higher testosterone concentrations in maternal plasma during gestation (27, 41), more recent findings suggest that there is no significant difference (42–45). Based on more recent evidence, we believe that the fetal sex differences we have observed for testosterone are not due to elevated fetal testosterone in males impacting maternal testosterone. Nevertheless, it remains unclear what biological mechanisms may be differentially at play between fetal sexes. One group has proposed that high concentrations of testosterone may act on the endometrium to disrupt its production of secretory proteins, which may result in elevated risk for preterm delivery (8), but how this process may differ between male and female fetuses is unknown.

### Thyroid Hormones

Decreased odds of PTB have been shown with increased concentrations of fT4 in the second (24) and third (46) trimesters, which contradicts our finding that fT4 was inversely associated with gestational age at birth and positively associated with odds of overall and spontaneous PTB. One prior study also found increased odds of PTB with greater T3 concentrations at 10 and 26 weeks gestation (46). Similarly, we found that T3 was associated with increased odds of spontaneous PTB when the fetus was male. Mechanisms of the association between thyroid hormones and PTB are poorly understood, but previous research has suggested that altered thyroid hormone concentrations may be involved in other disease states or exposures for which we have evidence of associations with PTB, such as oxidative stress and inflammation (47–49), or environmental exposures such as phthalates (50–52).

Thyroid hormones are critical for proper brain and skeletal development of the fetus (13). The fetus requires maternal thyroid hormones in the first half of pregnancy, during which time the fetal thyroid gland is immature and cannot produce its own hormones (14). During the second half of pregnancy, both maternal and fetal thyroid hormones are at play. Thyroid hormones cross the placenta *via* thyroid hormone transporters (15), and differential expression of these transporters can affect the levels of thyroid hormones available to the fetus. Thus, assessment of expression patterns of hormone transporters may be necessary to fully understand thyroid hormone associations with timing of labor.

Several previous studies have observed that male fetal sex is associated with a greater risk of delivering preterm. Proposed biological explanations for this observation include a pro-inflammatory environment generated by a male fetus (25) and larger size at birth for males relative to females (26). We observed significant associations with timing of labor unique to women carrying a male fetus for CRH, estriol, progesterone, and fT4, providing further evidence that the effect of fetal sex on the timing of labor is complex, possibly involving diverse endocrine pathways.

### Strengths and Limitations

The present study was subject to several limitations. We were not able to measure hCG, for which previous work has suggested a fetal sex difference is present (53). We also did not assess thyroid autoantibody status which could be clouding our thyroid hormone results due to unmeasured confounding. Some critical changes in the maternal endocrine environment occur later in gestation than we were able to measure, such as the exponential increase in CRH right before the onset of labor. Although the goal of this study was to determine whether mid-pregnancy hormone levels were indicative of increased risk of adverse pregnancy outcomes, measurements at later time points could shed additional light on the various endocrine pathways implicated in adverse birth outcomes. We observed low rates of the spontaneous subtype of PTB, which reduces the reliability of effect estimates, particularly when investigating effects specific to study visit and fetal sex. However, these lower rates may have been observed because we excluded women with preexisting conditions from our cohort to allow more precise examination of associations between hormone concentrations and birth outcomes, since preexisting conditions can influence hormone concentrations and susceptibility to adverse birth outcomes. Furthermore, excluding women with preexisting conditions may limit the generalizability of our findings.

Despite the aforementioned limitations, this study was also strong in various ways. This is one of few studies to assess a broad panel of hormone concentrations in relation to gestational age, which is advantageous because the disease state may implicate multiple endocrine pathways. This is also one of few studies to assess hormones at more than one time point during gestation to investigate different windows of susceptibility to hormonal disruption. Assessment of hormones at multiple time points is also necessary for hormones whose concentrations change dramatically through pregnancy, including CRH, estriol, and progesterone. We are also one of few groups to assess interactions between gestational hormone concentrations and fetal sex, despite existing research suggesting differential susceptibility to early delivery between male and female fetuses. Finally, our study was strengthened by a higher sample size of mothers than was seen in most previously published cohorts, which is particularly important when studying rare outcomes such as spontaneous PTB.

In conclusion, we observed a range of associations between hormones and timing of delivery. We found differences based on the timing of hormone assessment, and many significant findings were unique to mothers carrying a male fetus. Future work will attempt to place these findings in the context of relevant environmental contaminants on the island of Puerto Rico by exploring possibilities of endocrine disruption as a mediator between chemical exposures and pregnancy outcomes. Additional studies are needed to more fully elucidate the role of altered hormone concentrations in the etiology of adverse birth outcomes.

## Data Availability Statement

The raw data supporting the conclusions of this article will be made available by the authors, without undue reservation.

## Ethics Statement

This study was approved by the research and ethics committees of the University of Michigan School of Public Health, University of Puerto Rico, Northeastern University, and participating hospitals and clinics. The patients/participants provided their written informed consent to participate in this study.

## Author Contributions

AC analyzed and interpreted data and drafted the original article. JM, JC, AA, RL-C, and CV made substantial contributions to the conception and design of the study. DW and ZR contributed to acquisition of data. BM, RL-C, and MO’n assisted with interpretation of data. All authors contributed to the article and approved the submitted version.

## Funding

This work was supported by grant number P42ES017198 from the NIH; grant numbers R01ES031591, R01ES032203, and P30ES017885 from the NIEHS; and program grant number UH3OD023251 for the Environmental influences on Child Health Outcomes (ECHO) from the NIH, Office of the Director.

## Conflict of Interest

The authors declare that the research was conducted in the absence of any commercial or financial relationships that could be construed as a potential conflict of interest.

## Publisher’s Note

All claims expressed in this article are solely those of the authors and do not necessarily represent those of their affiliated organizations, or those of the publisher, the editors and the reviewers. Any product that may be evaluated in this article, or claim that may be made by its manufacturer, is not guaranteed or endorsed by the publisher.
