# Supplementary material for: Gestational Hormone Concentrations Are Associated With Timing of Delivery in a Fetal Sex-Dependent Manner
Source: Front Endocrinol (Lausanne). 2021 Sep 15;12:742145. doi: 10.3389/fendo.2021.742145 (PMC8479114; doi:10.3389/fendo.2021.742145)
Supplement: Supplementary file 1 [file Table_1.docx]

| **Supplementary Table S1. Distributions of gestational average (GA) and visit specific hormone concentrations** | | | | | | | | | | | | | |
| --- | --- | --- | --- | --- | --- | --- | --- | --- | --- | --- | --- | --- | --- |
|  |  | **N** | **min** | **25th** | **50th** | **75th** | **90th** | **95th** | **Max** | **Geo. Mean** | **Geo. Stdv** | **IQR** | **ICC (95% CI)** |
| **CRH (pg/mL)** | GA | 976 | 3.50 | 15.4 | 43.2 | 86.3 | 118 | 148 | 243 | 35.7 | 2.77 | 70.9 | 0.71 (0.66, 0.74) |
|  | 18wks | 818 | 3.50 | 15.1 | 37.6 | 84.3 | 121 | 156 | 254 | 34.4 | 2.89 | 69.2 |  |
|  | 26wks | 602 | 3.50 | 14.7 | 39.3 | 88.2 | 130 | 159 | 249 | 34.2 | 2.95 | 73.4 |  |
| **Estriol (mg/mL)** | GA | 971 | 0.74 | 15.6 | 23.1 | 33.0 | 44.7 | 57.5 | 265 | 22.7 | 1.80 | 17.4 | -0.22 (-0.35, -0.11) |
|  | 18wks | 812 | 0.74 | 11.3 | 15.1 | 22.2 | 31.8 | 41.5 | 108 | 15.8 | 1.75 | 10.9 |  |
|  | 26wks | 600 | 6.90 | 29.3 | 38.2 | 50.5 | 64.4 | 74.6 | 265 | 38.7 | 1.55 | 21.2 |  |
| **SHBG (pg/mL)** | GA | 976 | 47.6 | 413 | 538 | 668 | 818 | 895 | 1404 | 522 | 1.45 | 254 | 0.76 (0.72, 0.79) |
|  | 18wks | 820 | 47.6 | 389 | 516 | 630 | 775 | 850 | 1461 | 491 | 1.47 | 241 |  |
|  | 26wks | 602 | 123 | 434 | 566 | 723 | 898 | 979 | 1428 | 558 | 1.45 | 289 |  |
| **Progesterone (ng/mL)** | GA | 973 | 10.1 | 36.6 | 50.4 | 71.0 | 99.4 | 124 | 1037 | 51.8 | 1.68 | 34.5 | 0.07 (-0.04, 0.17) |
|  | 18wks | 815 | 10.1 | 29.2 | 39.3 | 54.5 | 71.9 | 85.0 | 301 | 40.1 | 1.59 | 25.3 |  |
|  | 26wks | 601 | 19.4 | 51.2 | 73.5 | 104 | 146 | 179 | 1037 | 74.4 | 1.70 | 53.2 |  |
| **TSH (uIU/mL)** | GA | 971 | 0.03 | 0.71 | 1.10 | 1.72 | 2.38 | 2.99 | 32.4 | 1.08 | 1.96 | 1.02 | 0.72 (0.67, 0.75) |
|  | 18wks | 812 | 0.02 | 0.67 | 1.05 | 1.66 | 2.38 | 2.88 | 40.9 | 1.03 | 2.06 | 0.99 |  |
|  | 26wks | 600 | 0.11 | 0.72 | 1.15 | 1.75 | 2.43 | 3.23 | 25.7 | 1.12 | 1.96 | 1.03 |  |
| **fT4 (ng/dL)** | GA | 976 | 0.11 | 1.09 | 1.62 | 2.02 | 2.32 | 2.50 | 8.35 | 1.41 | 1.68 | 0.93 | 0.75 (0.71, 0.79) |
|  | 18wks | 818 | 0.11 | 1.03 | 1.57 | 2.01 | 2.30 | 2.48 | 8.35 | 1.34 | 1.84 | 0.98 |  |
|  | 26wks | 602 | 0.11 | 1.10 | 1.61 | 2.03 | 2.33 | 2.49 | 4.68 | 1.39 | 1.75 | 0.93 |  |
| **T4 (ug/dL)** | GA | 975 | 0.35 | 0.89 | 1.00 | 1.10 | 1.21 | 1.28 | 1.72 | 0.99 | 1.19 | 0.21 | 0.65 (0.59, 0.69) |
|  | 18wks | 818 | 0.35 | 0.90 | 1.01 | 1.12 | 1.21 | 1.28 | 1.72 | 1.00 | 1.19 | 0.22 |  |
|  | 26wks | 602 | 0.44 | 0.83 | 0.96 | 1.08 | 1.19 | 1.23 | 1.43 | 0.94 | 1.21 | 0.25 |  |
| **T3 (mg/mL)** | GA | 971 | 6.20 | 10.5 | 11.8 | 13.2 | 14.4 | 15.2 | 19.0 | 11.7 | 1.18 | 2.70 | 0.72 (0.67, 0.75) |
|  | 18wks | 812 | 6.80 | 10.6 | 11.9 | 13.3 | 14.4 | 15.3 | 19.0 | 11.8 | 1.19 | 2.70 |  |
|  | 26wks | 600 | 5.30 | 10.3 | 11.6 | 13.0 | 14.2 | 14.9 | 20.6 | 11.5 | 1.19 | 2.75 |  |
| **Testosterone (pg/mL)** | GA | 973 | 2.80 | 53.0 | 107 | 557 | 819 | 992 | 2868 | 160 | 3.55 | 504 | 0.86 (0.83, 0.88) |
|  | 18wks | 815 | 1.10 | 50.1 | 105 | 544 | 789 | 952 | 2500 | 156 | 3.66 | 493 |  |
|  | 26wks | 601 | 9.20 | 59.3 | 121 | 650 | 933 | 1092 | 3291 | 185 | 3.64 | 591 |  |
| Gestational average values were calculated as arithmetic means for normally distributed hormones and geometric means for log-normally distributed hormones. | | | | | | | | | | | | | |

| **Supplementary Table S2**. **Differential associations between birth outcomes and hormones measured at 18 and 26 weeks by fetal sex.** | | | | | |
| --- | --- | --- | --- | --- | --- |
|  | **Gestational Age at Birth (days)** | | | | |
|  | **Female pregnancies** | |  | **Male pregnancies** | |
|  | **18 weeks** | **26 weeks** |  | **18 weeks** | **26 weeks** |
|  | **OR (95% CI)** | **OR (95% CI)** | **Int P** | **OR (95% CI)** | **OR (95% CI)** |
| **CRH** | -1.26 (-3.71, 1.12) | -0.7 (-3.22, 1.89) | 0.319 | -2.73 (-4.97, -0.42) | -2.1 (-4.62, 0.42) |
| **Estriol** | 0.07 (-2.24, 2.31) | 3.92 (0.77, 7.07) | 0.125 | -1.96 (-4.41, 0.49) | 1.96 (-1.19, 5.18) |
| **Progesterone** | -2.1 (-4.27, 0.14) | 1.12 (-1.19, 3.43) | 0.015 | -4.9 (-7.07, -2.73) | -1.68 (-3.78, 0.42) |
| **Prog/E3** | -1.12 (-2.8, 0.63) | -1.61 (-3.71, 0.49) | 0.542 | -1.75 (-3.5, -0.07) | -2.24 (-4.27, -0.28) |
| **Testosterone** | -0.49 (-3.29, 2.31) | -0.77 (-3.85, 2.24) | 0.121 | 2.03 (-0.63, 4.62) | 1.68 (-1.26, 4.62) |
| **SHBG** | -0.84 (-2.73, 1.05) | -1.12 (-3.29, 1.05) | 0.510 | -0.07 (-1.96, 1.75) | -0.35 (-2.45, 1.68) |
| **TSH** | 0.49 (-1.26, 2.31) | -0.56 (-2.66, 1.47) | 0.914 | 0.42 (-1.4, 2.17) | -0.7 (-2.8, 1.33) |
| **T3** | -0.42 (-2.59, 1.82) | 0.84 (-1.68, 3.36) | 0.281 | -1.82 (-3.92, 0.28) | -0.56 (-2.87, 1.68) |
| **fT4** | -1.33 (-3.22, 0.56) | -0.07 (-2.31, 2.1) | 0.245 | -2.73 (-4.76, -0.7) | -1.47 (-3.57, 0.63) |
| **T4** | -1.05 (-2.94, 0.91) | -0.91 (-2.94, 1.19) | 0.653 | -1.54 (-3.5, 0.42) | -1.4 (-3.57, 0.77) |
| **T3/T4** | 0.07 (-2.03, 2.24) | 1.54 (-0.84, 3.92) | 0.260 | -1.33 (-3.36, 0.7) | 0.14 (-2.03, 2.31) |
|  | **Preterm Birth** | | | | |
|  | **Female pregnancies** | |  | **Male pregnancies** | |
|  | **18 weeks** | **26 weeks** |  | **18 weeks** | **26 weeks** |
|  | **OR (95% CI)** | **OR (95% CI)** | **Int P** | **OR (95% CI)** | **OR (95% CI)** |
| **CRH** | 0.68 (0.40, 1.15) | 0.64 (0.37, 1.13) | 0.002 | 1.82 (1.09, 3.05) | 1.73 (0.98, 3.05) |
| **Estriol** | 0.92 (0.56, 1.51) | 0.52 (0.25, 1.11) | 0.022 | 1.81 (1.07, 3.06) | 1.03 (0.53, 2.01) |
| **Progesterone** | 0.95 (0.60, 1.52) | 0.74 (0.42, 1.30) | 0.011 | 1.88 (1.16, 3.04) | 1.46 (0.96, 2.23) |
| **Prog/E3** | 1.00 (0.67, 1.48) | 1.36 (0.85, 2.17) | 0.880 | 0.96 (0.67, 1.39) | 1.31 (0.84, 2.04) |
| **Testosterone** | 2.21 (1.16, 4.23) | 2.15 (1.08, 4.27) | 0.000 | 0.52 (0.30, 0.89) | 0.50 (0.26, 0.96) |
| **SHBG** | 0.72 (0.49, 1.07) | 0.60 (0.37, 0.96) | 0.032 | 1.22 (0.82, 1.83) | 1.01 (0.66, 1.55) |
| **TSH** | 0.90 (0.61, 1.32) | 1.24 (0.78, 1.97) | 0.397 | 1.10 (0.75, 1.60) | 1.52 (0.98, 2.36) |
| **T3** | 0.66 (0.41, 1.06) | 0.74 (0.42, 1.30) | 0.013 | 1.35 (0.86, 2.13) | 1.53 (0.95, 2.46) |
| **fT4** | 1.08 (0.71, 1.66) | 0.97 (0.59, 1.58) | 0.115 | 1.63 (1.06, 2.51) | 1.46 (0.93, 2.30) |
| **T4** | 1.13 (0.74, 1.71) | 1.06 (0.67, 1.67) | 0.397 | 1.39 (0.93, 2.08) | 1.31 (0.83, 2.08) |
| **T3/T4** | 0.66 (0.41, 1.04) | 0.71 (0.42, 1.19) | 0.032 | 1.18 (0.77, 1.81) | 1.27 (0.81, 1.99) |
|  | **Spontaneous Preterm Birth** | | | | |
|  | **Female pregnancies** | |  | **Male pregnancies** | |
|  | **18 weeks** | **26 weeks** |  | **18 weeks** | **26 weeks** |
|  | **OR (95% CI)** | **OR (95% CI)** | **Int P** | **OR (95% CI)** | **OR (95% CI)** |
| **CRH** | 0.75 (0.38, 1.49) | 0.57 (0.27, 1.20) | 0.003 | 2.73 (1.38, 5.43) | 2.10 (0.96, 4.58) |
| **Estriol** | 1.22 (0.64, 2.33) | 1.35 (0.51, 3.57) | 0.340 | 1.73 (0.92, 3.25) | 1.92 (0.79, 4.64) |
| **Progesterone** | 1.16 (0.65, 2.07) | 1.70 (0.85, 3.38) | 0.303 | 1.60 (0.89, 2.86) | 2.34 (1.36, 4.03) |
| **Prog/E3** | 0.85 (0.51, 1.41) | 1.65 (0.88, 3.09) | 0.979 | 0.86 (0.54, 1.36) | 1.67 (0.93, 3.00) |
| **Testosterone** | 1.58 (0.69, 3.62) | 1.61 (0.64, 4.02) | 0.001 | 0.31 (0.15, 0.64) | 0.31 (0.13, 0.79) |
| **SHBG** | 0.99 (0.58, 1.69) | 0.98 (0.51, 1.90) | 0.418 | 1.29 (0.78, 2.16) | 1.28 (0.71, 2.32) |
| **TSH** | 0.71 (0.46, 1.12) | 0.93 (0.50, 1.75) | 0.265 | 0.99 (0.63, 1.58) | 1.30 (0.73, 2.30) |
| **T3** | 0.78 (0.42, 1.43) | 0.79 (0.37, 1.69) | 0.013 | 2.01 (1.10, 3.65) | 2.05 (1.10, 3.84) |
| **fT4** | 1.48 (0.88, 2.52) | 1.55 (0.79, 3.03) | 0.577 | 1.79 (1.05, 3.07) | 1.87 (1.02, 3.42) |
| **T4** | 1.08 (0.63, 1.86) | 1.10 (0.60, 2.03) | 0.386 | 1.44 (0.87, 2.36) | 1.46 (0.79, 2.69) |
| **T3/T4** | 0.80 (0.45, 1.44) | 0.83 (0.41, 1.69) | 0.063 | 1.57 (0.91, 2.71) | 1.62 (0.90, 2.92) |
| CRH, estriol, SHBG, progesterone, TSH, testosterone, and prog/E3 were natural log transformed for analyses.  Effect estimates refer to an interquartile range increase in hormone concentration.  Int P indicates significance of effect modification by fetal sex – i.e. the p-value for the interaction term between hormone concentration and fetal sex indicator. | | | | | |
